# Success is not necessarily automatic

**DOI:** 10.1155/S1463924698000157

**Published:** 1998

**Authors:** Alastair B. Selkirk

**Affiliations:** Pharmaceutical and Analytical Research and Development Abbott Laboratories 1401 Sheridan Road North Chicago IL 60064-4000 USA

## Abstract

There are a number of factors (planning, process optimization, organizational structure, people development and the need to see the total picture) that must be in place for automation to be as effective as possible. This paper discusses these factors and their relationship with automation. It evaluates less obvious areas associated with automation, as well as the more established ones, and discusses the premise that it is the integration of all these aspects that truly offers the biggest opportunities.


Journal of Automatic Chemistry, Vol. 20, No. 4 (July-August 1998) pp. 109-110

### Success is not necessarily automatic

**Alastair B. Selkirk**
*Pharmaceutical and Analytical Research and Development, Abbott Laboratories, 1401 Sheridan Road, North Chicago, IL 60064-4000, USA*

*There are a number of factors (planning, process optimization, organizational structure, people development and the need to see the total picture) that must be in place for automation to be as effective as possible. This paper discusses these factors and their relationship with automation. It evaluates less obvious areas associated with automation, as well as the more established ones, and discusses the premise that it is the integration of all these aspects that truly offers the biggest opportunities.*

### Introduction

Over the last few years, the pharmaceutical industry has steadily become more competitive. Clear evidence of this is seen in the number of mergers and takeovers that have occurred in the last five to seven years. There is no reason to assume that this state of affairs will get anything but worse as we approach the next millennium. For companies to be successful, they need a source of breakthrough molecular entities, and a development process that permits the drug to move effectively from discovery through IND to NDA or equivalent.

Thus, many companies are re-engineering their development processes to achieve the latter and give them a competitive edge. These processes must be able to significantly reduce the development time, for example by 25–30%, while, at the very least, maintaining quality. They must be achieved without a burnout of staff, an event which unfortunately is becoming steadily more common in the industry.

To further exacerbate the problems, the complexity of the development process significantly increases each year, as do the registration requirements around the world. In the Chemistry, Manufacturing and Controls (CMC) area, the advent of ICH, SUPAC, PAIs, etc. have significantly changed the ways things are done, with far more planning and internal controls being applied. In the author's department, the concept of 'Good Scientific Practices' has been introduced to define all work outside GMP and GLP.

Finally, global integration is now essential for companies to be successful.

In many instances, it has been assumed that automation and computerization, the 'magic black box', can solve these problems. This paper proposes the hypothesis that automation alone will not provide the necessary improvements: indeed, such an approach may result in a decreased efficiency. It then reviews some of the factors contributing to the successful acceleration of the development process and evaluates the role of automation as an integral part of that accelerated process.

### Processes

The processes involved in developing a medicine from the glint in a chemist's eye through to launch are extremely complicated. Even restricting these to the CMC areas, they are still immensely complex. Partly as a result of that, and partly as a result of the way many of the processes have evolved, they are generally very inefficient.

A number of years ago, the author's previous company went through a major re-engineering exercise to reduce the time from IND to NDA. It was quickly decided that development time could be reduced by 50%. While this may not be possible across the board, it is likely that savings of 25–30% should be readily realized. So how do you achieve such process improvements?

First, question everything. There must be no sacred cows, and re-engineering is not for the faint-hearted. Then, prepare a flow diagram of how things really happen. This is often a real eye-opener with many processes going round and round in never-ending loops. Finally, get a completely clean piece of paper and design it from scratch as if you were a start-up company. This then brings you to the hardest part, implementation. The only advice here is do not give up. You will be very tempted to, as the revamped processes will probably be worse in the beginning and you will be surrounded by a lot of unhappy staff. Perservere, it will take time. Most of the re-engineering exercises that fail are because they were not seen through to completion.

### Planning

Planning is very important. For plans to work, they must include key performance indicators so that it can be determined whether a company is on plan or not. It also requires training. Training should be taken into teams on a just-in-time basis and should ask and answer the questions:

- Is the team on track?
- How do they know they are on track?
- How do they stay on track?
- How do they get back on track?

This kind of training has been shown to significantly increase work without any staff burnout.

### Organizational structure

Many companies make the mistake of designing their organizational structure and then designing the process to fit the organizational structure. The opposite is the way to efficiency. Since so much of the business in the pharmaceutical industry is cross-functional, then the organizational structure should be primarily cross-func-

0142-0453/98 $12·00 © 1998 Taylor & Francis Ltd tional. The greatest opportunities for improved efficiency are often not in how well a chemist analyses a sample or a formulator formulates a product, but, rather, in how well they interact with each other.

Real progress can be made at cross-functional interfaces. In my present company, we organized by setting up a number of project teams, which were essentially designed to be akin to small start-up companies. The teams would be fully accountable for the running of that project. Such an organization has two basic problems to overcome. The first is how to avoid reinventing the wheel for each new project. The second is how to stop the perception that staff will not have a job when the project finishes. Centres of Excellence were created which acted as 'homes' for people waiting to be assigned or reassigned to projects. In reality, these waiting periods were non-existent, but the centres provided a perceived safety net. In addition, a small core of people were kept in the centres. Their job was to set standards across the projects, define process improvements to improve project team effectiveness, and provide experts and mentors to the teams, i.e. to have the time and focus to implement the improvements that everybody knows need to be done, but nobody has the time to do.

### Technology

What has all this to do with automation? In far too many instances, there is a perception that there is a 'black box' answer to all our problems, a perception that we will develop a system and all that will be needed is to simply press a button. Technology can do much to support and facilitate improvement programmes, but, in itself, it does not solve them. Without good processes, well-designed and well-executed plans, supported by the correct organizational structure, it is simply the inefficient that is automated.

In our organization, we include a number of activities under the umbrella of automation. These include robotics, laboratory acquisition systems, laboratory management systems, and document management systems. The primary product that research and development manufactures is information, often in the form of documents. If information and documentation are effectively managed, then projects will also be effectively managed.

Thus, robotic systems, laboratory acqusition and management systems, etc. must be integrated in a cross-functional manner with document management systems to form an overall comprehensive system. Too often, they exist as separate islands of technology. To achieve this integration, an overall strategic plan has to be developed and then peeled like an onion with increasing detail at each stage. If the plan is not integrated, there is a real danger of simply shifting the bottlenecks, rather than achieving real progress.

The other major issue with technology development is the 'creeping sophistication syndrome'. Here, the system has more and more refinements planned and developed. The result is that it is never finished and never becomes fully operational.

### People

Since people are our most valuable resources, it is interesting to consider the effects of automation on them. Automation properly developed undoubtedly improves both consistency and productivity. There is a perception, however, that it decreases flexibility and innovation. The errors in this perception should be pointed out to the people involved very early in the process. Technology should be used to automate the results of innovation. Receptor screening is a good example of this. We should, however, also innovate the results of automation. Combinatorial chemistry is a classic example of this, where the creation of huge numbers of new molecules greatly increases the innovative opportunities, both in the discovery of new drugs and in the improvement of existing drugs. However, automation should not be allowed to stifle personal creativity—major discovery breakthroughs are still more likely to come from personal ideas. Such free thinking must be encouraged and automation used to improve its implementation.

Automation should be used to reduce bureaucracy. Here, the machines can be regulated rather than the people; people can be freed from repetitive jobs, which they are not usually very good at, to increase their innovative abilities.

### Conclusion

Automation can undoubtedly increase the efficiency of a process, be it a simple single process or a complex interaction of processes like those related to drug development. For automation to be fully effective, it needs to be part of an overall integrated system that also includes process improvement, management systems, organizational structure, and even a belief and culture that optimizes those opportunities provided by automation. Great strides have been made in the technology related to automation. I believe the biggest challenge we now face is in its integration. Automation improves efficiency, but we need to ensure it also optimizes effectiveness.

